# Controllable optical modulation of blue/green up-conversion fluorescence from Tm^3+^ (Er^3+^) single-doped glass ceramics upon two-step excitation of two-wavelengths

**DOI:** 10.1038/srep45650

**Published:** 2017-04-03

**Authors:** Zhi Chen, Shiliang Kang, Hang Zhang, Ting Wang, Shichao Lv, Qiuqun Chen, Guoping Dong, Jianrong Qiu

**Affiliations:** 1 State Key Laboratory of Luminescent Materials and Devices, and Guangdong Provincial Key Laboratory of Fiber Laser Materials and Applied Techniques, South China University of Technology, Guangzhou 510641, China; 2 Key Laboratory of Shock Wave and Detonation Physics, Institute of Fluid Physics, CAEP, Mianyang 621900, China; 3 College of Optical Science and Engineering, State Key Laboratory of Modern Optical Instrumentation, Zhejiang University, Hangzhou 310027, China

## Abstract

Optical modulation is a crucial operation in photonics for network data processing with the aim to overcome information bottleneck in terms of speed, energy consumption, dispersion and cross-talking from conventional electronic interconnection approach. However, due to the weak interactions between photons, a facile physical approach is required to efficiently manipulate photon-photon interactions. Herein, we demonstrate that transparent glass ceramics containing LaF_3_: Tm^3+^ (Er^3+^) nanocrystals can enable fast-slow optical modulation of blue/green up-conversion fluorescence upon two-step excitation of two-wavelengths at telecom windows (0.8–1.8 μm). We show an optical modulation of more than 1500% (800%) of the green (blue) up-conversion fluorescence intensity, and fast response of 280 μs (367 μs) as well as slow response of 5.82 ms (618 μs) in the green (blue) up-conversion fluorescence signal, respectively. The success of manipulating laser at telecom windows for fast-slow optical modulation from rear-earth single-doped glass ceramics may find application in all-optical fiber telecommunication areas.

As the internet applications continue to develop at an extremely fast pace, the dominant electronic interconnection approach suffers from issues of bandwidth and loss due to its performance restrictions in terms of speed, energy consumption, dispersion and cross-talking. Photonic technologies are central to our information-based society. Among photonic technologies, optical modulation is one of the most essential operations, which offers intrinsic advantages of higher bandwidth and lower loss^1^. Therefore, substantial research efforts are being directed towards optical modulation to exploit compact, cost-effective, efficient, fast and broadband light modulators for high-performance optical interconnects^2^.

In recent years, intense research efforts on optical modulation have concentrated on finding the fast and highly nonlinear media, including graphene and other two-dimensional layered materials^3^,^4^,^5^,^6^,^7^,^8^,^9^,^10^, gain nonlinear active media^11^, carrier-induced nonlinear semiconductor photonic crystal cavities^12^, nonlinear metals, semiconductors and low-dimensional carbon^13^, and optomechanical and phase-change metamaterials^14^,^15^. However, such materials suffer from defects of difficult fabrication, low production, high expense, low chemical durability, and are detrimental to environment^1^,^16^. On the contrary, rare-earth (RE) ions doped glass ceramics (GCs) can overcome these drawbacks. Besides, the well-engineered RE-doped GCs, combining the merits of glass (low expense, easy fabrication, good homogeneity and optical transparency) and crystals (high chemical durability and mechanical strength, intense crystal field effect)^17^,^18^,^19^,^20^, can be effortless for fiber drawing and will greatly impact the application in future all-optical fiber telecommunication^2^,^21^,^22^. As is known to all, photons interact weakly with each other, which requires the mediation of a physical system to produce efficient photon-photon interactions^16^,^23^,^24^,^25^. RE^3+^ ions possess vast amounts of energy levels, which provide convenience for us to realize optical modulation by adopting facile physical approaches^26^,^27^,^28^,^29^,^30^,^31^,^32^. Moreover, tunable excitation and emission wavelengths from visible to near-infrared (NIR) of RE^3+^-doped GCs guarantee optical modulation operating at telecom windows or at the visible range for emerging Li-Fi technology, showing notable advantages for optical data transmission systems^1^,^33^. Nevertheless, for a long time, fast-slow optical modulation of up-conversion (UC) fluorescence from RE^3+^ ion single-doped GCs by utilizing a strategy named “two-step excitation of two-wavelengths” has been overlooked^34^,^35^. The inclusion of electronic structure of the single-doping RE^3+^ ion has been argued to be particularly advantageous to manipulate the speed of electrons populated fully in the excited state^29^,^36^, by controlling one ground state absorption (GSA) or excited state absorption (ESA) wavelength laser as a gating beam combined simultaneously with another continuous-wave (C.W.) ESA or GSA wavelength laser beam, which in turn affect the optical switching “on-off” response. Hence, it gives an efficacious physical method for the realization of fast-slow optical modulation of UC fluorescence.

In this work, we introduce an approach for future all-optical information processing using two-step excitation of two-wavelengths at telecom windows from germanate oxyfluoride GCs containing LaF_3_: Tm^3+^ (Er^3+^) nanocrystals, which enables optical modulation of blue/green UC fluorescence with fast-slow response. The optical switching “on-off” response is relatively fast by manipulating the ESA wavelength laser as gating beam coupled simultaneously with a C. W. laser beam of GSA wavelength for both Tm^3+^ and Er^3+^ single-doped GCs. Conversely, the “on-off” response becomes much slower through modulating the GSA wavelength laser as gating beam combined simultaneously with a C. W. laser beam of ESA wavelength. Furthermore, we put insight into the mechanism responsible for this fast-slow optical modulation, which reveals the presence of differentiation of the speed of electrons populated fully in the excited state manipulated by various pumping tactic^29^,^36^. Importantly, the success of manipulating light at telecom windows for fast-slow optical modulation of blue/green UC fluorescence from LaF_3_:Tm^3+^ (Er^3+^) nanocrystals embedded germanate oxyfluoride GCs has been empowered by imaginative designs, which may provide powerful opportunities for novel all-optical fiber data processing in future optical telecommunication fields^37^,^38^.

## Results

### Theory, design, and concept for fast-slow UC fluorescence modulation

**Figure 1:**
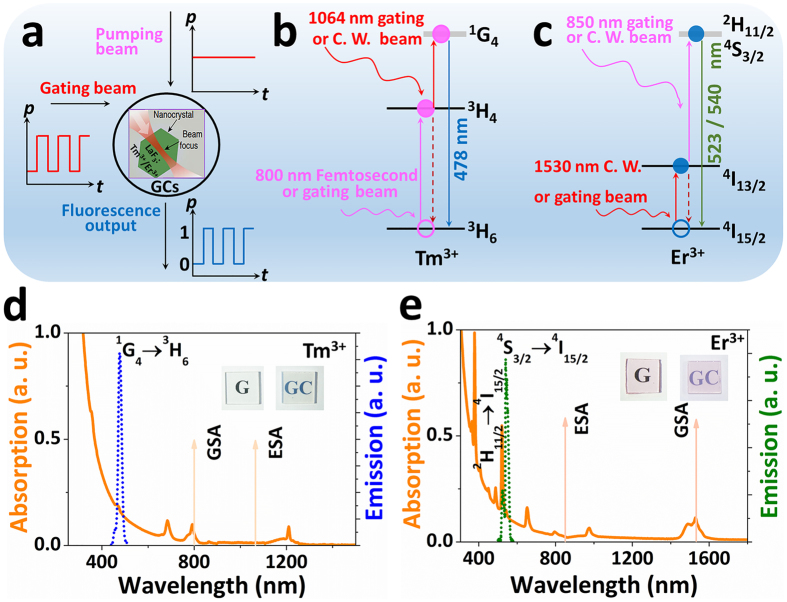
Concept of fast-slow optical modulation of blue/green UC fluorescence from Tm^3+^ (Er^3+^) single-doped GCs upon two-step excitation of two-wavelengths. (**a**) The emitted signal of the blue/green UC fluorescence is controlled by a modulated gating NIR laser coupled with a C. W. laser. (**b**,**c**) Schematic diagram of the electronic transitions involved in the fast-slow fluorescence modulation from Tm^3+^ (**b**) or Er^3+^ (**c**) single-doped GCs. (**d**,**e**) The absorption (solid orange line), and emission (dotted line) spectra of Tm^3+^ (**d**) or Er^3+^ (**e**) single-doped GCs. The vertical arrows indicate the locations of the GSA and ESA wavelengths. Insets: Photographs of the corresponding Tm^3+^ (**d**) or Er^3+^ (**e**) single-doped glass and GCs.

As sketched in Fig. 1(a–c), the concept of fast-slow optical modulation via two-step excitation of two-wavelengths is proposed. To validate this judicious design, the well-engineered Tm^3+^ (Er^3+^) single-doped GCs with lower optical losses (12.87 dB/cm at 800 nm and 3.64 dB/cm at 1064 nm for Tm^3+^ doped GCs, and 4.65 dB/cm at 850 nm and 8.22 dB/cm at 1530 nm for Tm^3+^ doped GCs. See Supplementary Material, Figs S1–S5, Table S1) were selected to study the fast-slow optical modulation properties. The room-temperature absorption and emission spectra of the Tm^3+^ (Er^3+^) single-doped GCs are shown in Fig. 1(d–e). The first GSA occurs at a wavelength of 800 nm for Tm^3+^ and 1530 nm for Er^3+^, which is a hallmark of the ^3^H_6_ → ^3^H_4_ and ^4^I_15/2_ → ^4^I_13/2_ transition of the Tm^3+^ and Er^3+^ single dopants, respectively^29^,^31^,^36^. The UC fluorescence emission can be triggered and expedited while selectively pumping with another non-resonant NIR laser through an efficient ESA step (Here, there’s no other ESA processes involved in this pumping strategy showing in Fig. S6). For the realization of this fast-slow optical modulation, we demonstrated a facile approach to controlling the speed of electrons populated fully in the excited state through selectively adjusting the NIR laser of GSA wavelength as C. W. or gating beam.

### Fast-slow optical modulation features for the blue/green UC fluorescence

**Figure 2:**
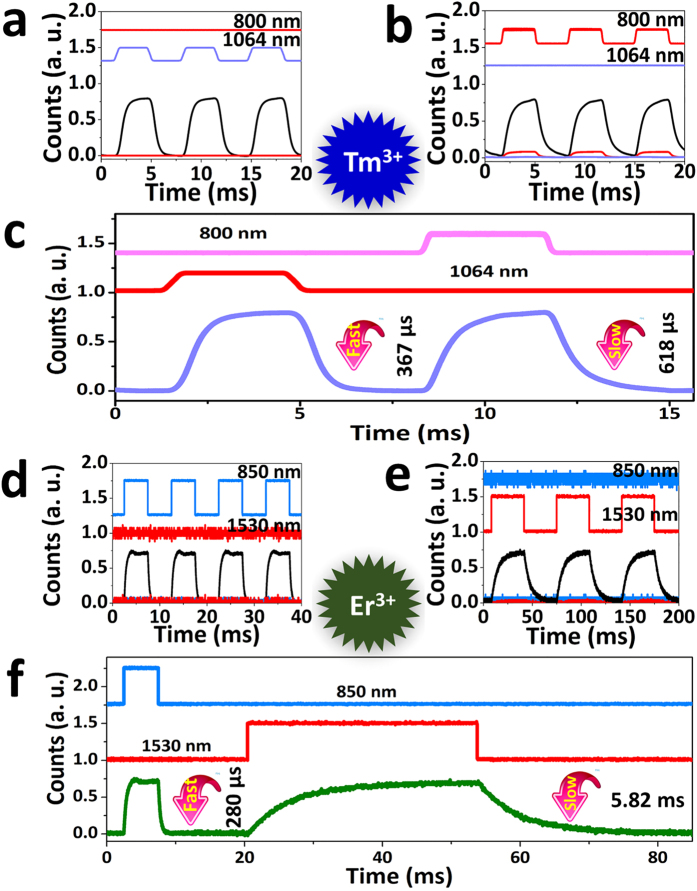
Fast-slow “on-off” optical modulation of blue/green UC fluorescence from Tm^3+^ (Er^3+^) single-doped GCs upon two-step excitation of two-wavelengths. (**a,b**) Continuous “on-off” cycling of the blue UC fluorescence from Tm^3+^ single-doped GCs with 800 nm (33.37 KW/cm^2^) and 1064 nm (3.65 MW/cm^2^) laser. The fluorescence signal follows the modulation of 1064 nm (**a**) or 800 nm laser (**b**), and negligible blue UC fluorescence signal is detected with only single 800 nm laser switched on (red lines in (**b**)). (**c**) Time-dependent fluorescence of Tm^3+^ single-doped GCs following repeated pulse sequence of 1064 and 800 nm laser light (upper panel in (**c**)). (**d,e**) Continuous “on-off” cycling of the green UC fluorescence from Er^3+^ single-doped GCs with 1530 nm (22.88 KW/cm^2^) and 850 nm (3.57 MW/cm^2^) laser. The fluorescence signal follows the modulation of 850 nm (**d**) or 1530 nm laser (**e**), and negligible green UC fluorescence signal is detected with only single 1530 nm laser switched on (red lines in (**e**)). (**f**) Time-dependent fluorescence of Er^3+^ single-doped GCs following repeated pulse sequence of 850 and 1530 nm laser (upper panel in (**f**)). The fluorescence decay time is fitted with the single exponential function: 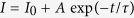, where I and I_0_ are the fluorescence intensity at time t and 0, A is constant, t is the time, and τ is the fluorescence decay time for the exponent^39^,^40^.

Figure 2 demonstrates fatigue-free switching of the blue/green UC fluorescence from Tm^3+^ (Er^3+^) single-doped GCs by two-step excitation of two-wavelengths. Using a home-built coaxial optical setup, bright UC fluorescence signal is detectable only when irradiating with both of two wavelengths laser, while single-wavelength laser irradiation alone cannot elicits a strong signal (Fig. S7). For the fast-slow optical modulation of blue fluorescence, we continuously excited Tm^3+^ single-doped GCs with 33.37 KW/cm^2^ of 800 nm laser, and periodically added 3.65 MW/cm^2^ of 1064 nm laser (Fig. 2(a)). The “on-off” cycling is reproducible and follows the modulation of 1064 nm laser, showing a faster response of 367 μs (Fig. 2(c)). Instead, when continuously excited Tm^3+^ single-doped GCs with 3.65 MW/cm^2^ of 1064 nm laser and periodically added 33.37 KW/cm^2^ of 800 nm laser (Fig. 2(b)), the “on-off” cycling is reproducible and follows the modulation of the 800 nm laser, showing a slower response of 618 μs (Fig. 2(c)). Vice versa for the fast-slow optical modulation of green fluorescence, we continuously excited Er^3+^ single-doped GCs with 22.88 KW/cm^2^ of 1530 nm laser, and periodically added 3.57 MW/cm^2^ of 850 nm laser (Fig. 2(d)), the “on-off” cycling is reproducible and follows the modulation of 850 nm laser, showing a faster response of 280 μs (Fig. 2(f)). Conversely, when continuously excited Er^3+^ single-doped GCs with 3.57 MW/cm^2^ of 850 nm laser and periodically added 22.88 KW/cm^2^ of 1530 nm laser (Fig. 2(e)), the “on-off” cycling is reproducible and follows the modulation of 1530 nm laser, showing an extremely slower response of 5.82 ms (Fig. 2(f)). Intriguingly, both of the fast-slow optical modulation can be performed, and the differentiation for the fast-slow response of green fluorescence modulating is as high as an order of magnitude.

### Blue/green UC fluorescence and efficiency manipulation

**Figure 3:**
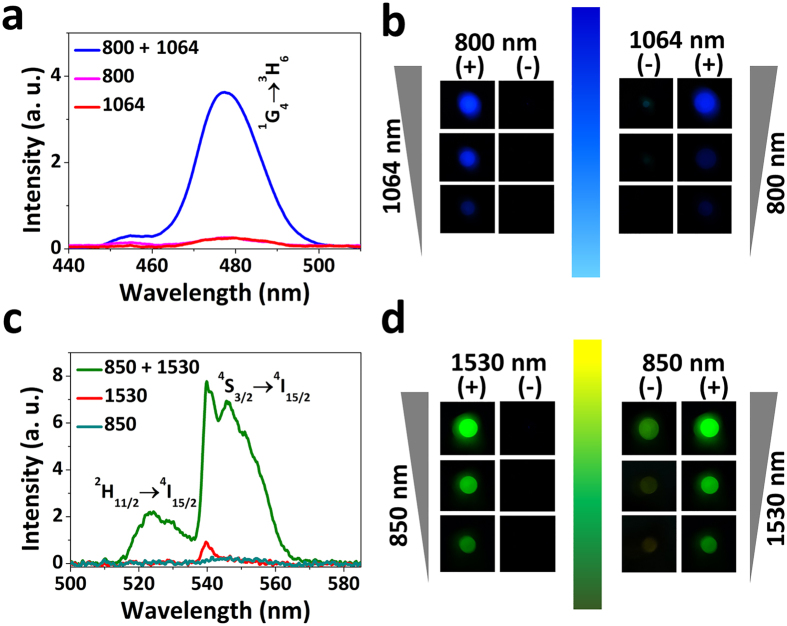
UC fluorescence spectra of Tm^3+^ (**a**) and Er^3+^ (**c**) single-doped GCs at room temperature following single-wavelength excitation, and two-step excitation of two-wavelengths. Fluorescence images of Tm^3+^ (**b**) and Er^3+^ (**d**) single-doped GCs with and without one NIR laser power fixed and increasing power of another NIR laser marked by arrows. “+” denotes excitation combined with another NIR laser of fixed power. “−” denotes excitation combined without another NIR laser.

**Figure 4:**
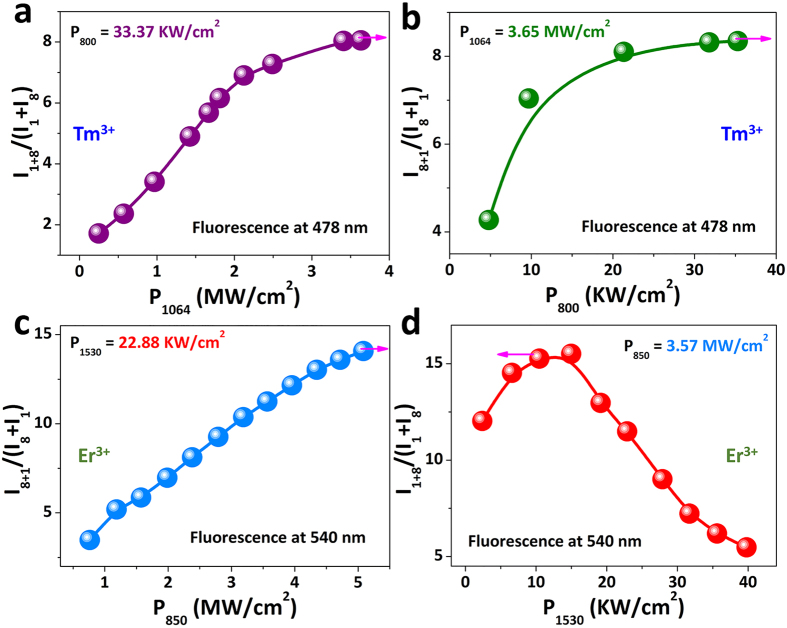
Possibility of modulating the blue/green UC fluorescence emission from the Tm^3+^ (**a,b**) and Er^3+^ (**c,d**) single-doped GCs. Setting the excitation power of one NIR laser to a fixed value while increasing the power of another NIR laser, a I_TSTW_/(I_SW1+_I_SW2_) fluorescence increase is observed (Where I_TSTW_, I_SW1_ and I_SW2_ represent the blue/green UC fluorescence intensities generated by two-step excitation of two-wavelengths, and by single-wavelength excitation. For Tm^3+^, I_TSTW_ denotes I_1+8_ in (**a**) and I_8+1_ in (**b**) upon two-step excitation of 1064 and 800 nm, I_SW1_ illustrates I_1_ in (**a**) and (**b**) upon single 1064 nm excitation, and I_SW2_ illustrates I_8_ in (**a**) and (**b**) upon single 800 nm excitation, respectively. For Er^3+^, I_TSTW_ denotes I_8+1_ in (**c**) and I_1+8_ in (**d**) two-step excitation of 850 and 1530 nm, I_SW1_ illustrates I_1_ in (**c**) and (**d**) upon single 1530 nm excitation, and I_SW2_ illustrates I_8_ in (**c**) and (**d**) upon single 850 nm excitation, respectively.), which is nonlinear when the NIR laser power increases^43^. The arrows represent the maximum tailorable UC fluorescence efficiency.

To validate the feasibility of the fast-slow optical modulation of blue/green UC fluorescence, we put insights into the performances of the UC fluorescence produced by two-step excitation of two-wavelengths. The co-irradiation by simultaneous two-wavelengths laser generates a notably enhanced UC emission spectrum from Tm^3+^ (Er^3+^) single-doped GCs (Fig. 3(a–c)), which is effortlessly distinguishable from that generated by single-wavelength excitation. The UC fluorescence emission intensity shows flexible modulating region. What’s more, the optimize UC fluorescence efficiency can be tuned over 800% for Tm^3+^ single-doped GCs and by up to 1500% for Er^3+^ single-doped GCs (Fig. 4(a–d)). In addition, the power of the output UC fluorescence can be tuned by changing the optical power of the input laser combined with and without another laser power fixed (Fig. 3(b–d)), where bright UC fluorescence can be obtained only by two-step excitation of two-wavelengths. The microscopic mechanisms of enhanced UC fluorescence tuned by two-step excitation of two-wavelengths has been shed light on the investigation for NIR laser power dependence of the fluorescence counts. As plotted in Fig. S8, using least-squares fitting^41^,^42^, only one photon is required for the blue/green UC fluorescence upon two-step excitation of two-wavelengths, which results in high efficient UC luminescence owing to the existing of an effective ESA process. Therefore, using different pumping methods through one NIR laser controlling another NIR laser, the blue/green UC fluorescence can be tailored for the exploitation of the “on-off” optical switching with fast-slow response.

### Dynamic evolution for the fast-slow optical modulation of blue/green UC fluorescence

**Figure 5:**
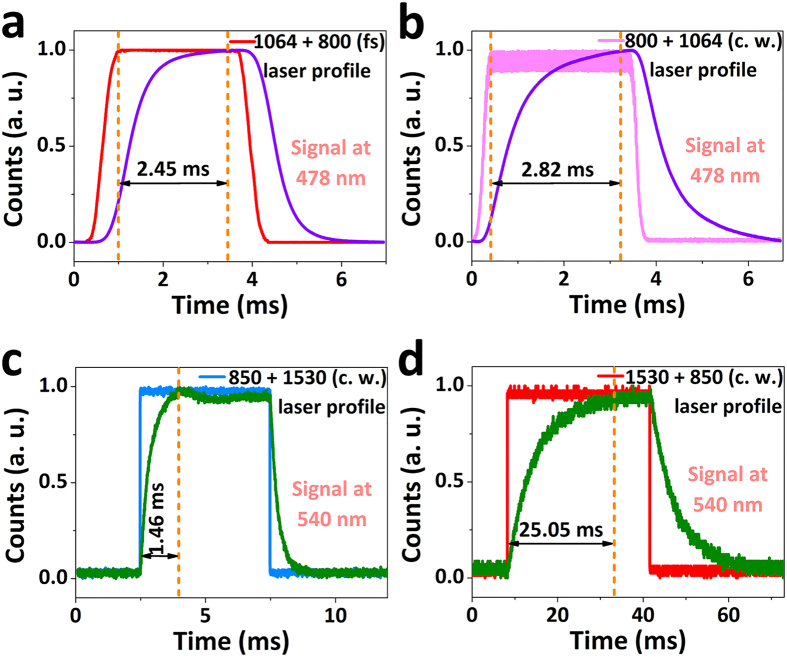
Time-dependent blue/green UC fluorescence intensity profiles of Tm^3+^ (**a,b**) and Er^3+^ (**c,d**) single-doped GCs under two-step excitation of one gating laser combined simultaneously with another C. W. laser with a fixed power. The rise time is defined as the time required from 10% to 90% of the output fluorescence, which can be recognized as non-steady state transition to steady state.

For a closer insight into the dynamic evolution processes of this fast-slow optical modulation, the kinetics of the UC fluorescence was thoroughly confirmed by time-resolved photoluminescence studies in Fig. 5. Under two-step excitation of 80 MHz 800 nm (as GSA wavelength) fs laser (that can be roughly recognized as a C. W. laser beam) combined simultaneously with 1064 nm (as ESA wavelength) gating laser with 150 Hz repetition frequency, the rise time is 2.45 ms to approach the steady-state for the blue UC fluorescence from the Tm^3+^ single-doped GCs (Fig. 5(a)). Instead, a little longer rise time of 2.82 ms is required to reach the steady-state for the blue UC fluorescence when tuning the GSA wavelength of 800 nm as gating laser beam at the same time changing the ESA wavelength of 1064 nm as C. W. laser beam (Fig. 5(b)). Quite surprisingly, in the case of optical modulation of green UC fluorescence from the Er^3+^ single-doped GCs, the rise time is just 1.46 ms to come to the steady-state upon two-step excitation of 1530 nm (as GSA wavelength) C. W. laser coupled simultaneously with 850 nm (as ESA wavelength) gating laser with 100 Hz repetition frequency (Fig. 5(c)). On the contrary, more than one order of magnitude rise time of 25.05 ms is needed for the steady-state under two-step excitation of 1530 nm (as GSA wavelength) gating laser combined simultaneously with 850 nm (as ESA wavelength) C. W. laser (Fig. 5(d)).

## Discussion

The full dependence of the fluorescence rate (F) for the fast-slow optical modulation upon two-step excitation of two-wavelengths is predicted by the rate-equation model (see Supplementary Material for details):


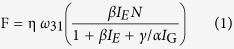


For the fast optical modulation,


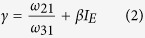


For the slow optical modulation,


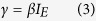


Where η is the collection efficiency of the detector, ω_31_ is intrinsic decay rates, N is the total numbers of ions, I_G_ is the GSA wavelength laser intensity, I_E_ is ESA wavelength laser intensity, α and β are the proportionality constants. The main assumption of the model is the excitation rates proportional to the power of laser, which is plausibly mediated by the speed of electrons populated fully in the first excited state rooting from the effect of GSA wavelength laser. For the fast optical modulation, the GSA wavelength laser is modulated as C. W. beam combined simultaneously with a gating laser of ESA wavelength. I_G_ is larger, I_E_ is lower and γ is lower in comparison with that for the slow optical modulation, resulting in the fast fluorescence rate of the “on-off” switching. Conversely, when the GSA wavelength laser is manipulated as gating beam coupled simultaneously with a C. W. laser of ESA wavelength, the I_G_ is lower, I_E_ is larger and γ is larger comparing with that for the fast optical modulation, leading to the appearance of slow fluorescence rate of the “on-off” switching. From the experimental observations we find excellent agreement with our analysis from the analytical solution of the model based on rate equation involving three energy levels, strongly verifying the feasibility of this fast-slow optical modulation. In addition, the investigation for fluorescence lifetime (Fig. S9), and time response of the switch affected by laser pulse duration (Fig. S10), can also provide proofs from dynamic evolution aspect for the fast-slow optical modulation of blue/green UC fluorescence.

In summary, we introduced an approach to future all-optical information processing using two-step excitation of two-wavelengths operating at telecom windows that enables fast-slow optical modulation of blue/green UC fluorescence from Tm^3+^ (Er^3+^) single-doped transparent GCs. We showed an optical modulation of more than 1500% (800%) of the green (blue) UC fluorescence intensity and a fast response of 280 μs (367 μs) as well as a slow response of 5.82 ms (618 μs) in the green (blue) UC fluorescence signal of LaF_3_:Tm^3+^ (Er^3+^) nanocrystals embedded germanate oxyfluoride GCs through two-step excitation of two-wavelengths. The study on dynamic evolution mechanism was indicated that the differentiation of the speed of electrons populated fully in the excited state manipulated by various pumping strategy was responsible for this fast-slow optical modulation. This fast-slow optical modulation of blue/green UC fluorescence from Tm^3+^ (Er^3+^) single-doped GCs was successfully manipulated by two-step excitation of two-wavelengths at telecom windows, which may provide a strategy for constructing all-optical fiber data processing in future optical telecommunication realms.

## Methods

### Fabrication of Samples

The preparations of germanate oxyfluoride GCs precipitating LaF_3_:Tm^3+^ (Er^3+^) nanocrystals are analogous to our previous works^31^. The precursor glasses, with a composition of 50GeO_2_-22Al_2_O_3_-13LaF_3_-15LiF-1XF_3_ (X = Tm or Er), were prepared at 1450 °C for 1 h by melt quenching technique. The precursor glasses were cut into blocks and heat-treated at 680 °C for 4 h to achieve GCs through crystallization. The samples were optically polished for further measurements of optical performances.

### Measurements and Characterization

X-ray diffraction (XRD) pattern of the samples were obtained on an X’Pert PRO X-ray diffractometer (PANalytical, Netherland) using Cu Kα (λ = 1.5418 Å) radiation, as shown in Fig. S1. A Lambda 900 spectrophotometer (PerkinElmer, USA) was employed to record the absorption spectra of the samples depicted in Fig. 2(d,e) and Fig. S2. The microstructures of the samples were analyzed by utilizing a high-resolution transmission electron microscope (HRTEM) 2100 F (JEOL, Japan), as illustrated in Figs S3 and S4. The optical loss of the GCs were measured by home-built optical setup with optical power meter PM320E (THORLABS, USA), as sketched in Fig. S5 and Table S1. The UC fluorescence spectra were recorded by a spectrometer HR4000 (Ocean Optics, USA).

### Optical Setup

**Figure 6:**
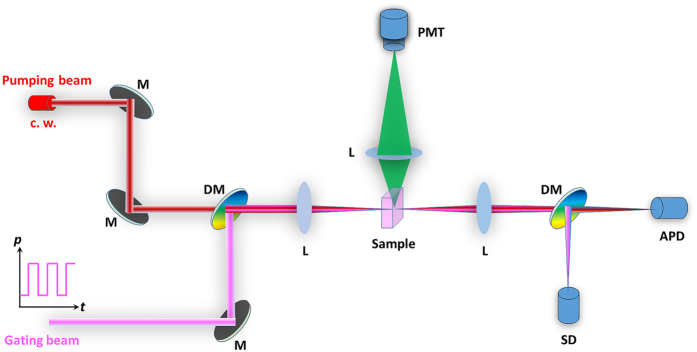
Optical setup. M: mirror; L: lens; DM: dichroic mirror; PMT: photomultiplier tube; SD: Si detector; APD: avalanche photodiode; C. W.: continuous-wave.

To investigate the fast-slow optical modulation of blue/green UC fluorescence, we used a coaxial optical path coupling two laser beams with dichroic mirror DMLP950 (THORLABS, USA) to illuminate samples at the confocal point, as sketched in Fig. 6. The size of the laser focal spot radius is determined by: ref. 34For Gauss laser beam:


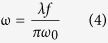


For monochromatic parallel laser beam:


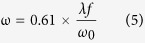


Where ω is laser focal spot radius, λ is the wavelength of the laser, f is the effective focal length of the lens, and ω_0_ is the entrance beam radius. The fluorescence signal is collected through vertical direction of the sample and sent to the photomultiplier tube (PMT) with high voltage of −500 V. The laser signal is detected by a Si detector (SD) or an avalanche photodiode (APD). For Tm^3+^, 1,064 nm laser (LEO Photoelectric, China) beam is superimposed with 80 MHz 800 nm femtosecond laser (COHERENT, USA) beam, after one of which passing through an optical chopper (THORLABS, USA) that allows us to temporally modulate its frequency and pulse width. For Er^3+^, 1530 nm laser beam (LEO Photoelectric, China) is superimposed with 850 nm laser (LEO Photoelectric, China) beam, after one of which using a signal-generation (Tektronix, USA) that allows us to temporally modulate its frequency and pulse width. The optical modulation signal and optical switching “on-off” response was collected with a TDS 3012B digital oscilloscope (Tektronix, USA). All the measurements were performed at room temperature.

## Additional Information

**How to cite this article**: Chen, Z. *et al*. Controllable optical modulation of blue/green up-conversion fluorescence from Tm^3+^ (Er^3+^) single-doped glass ceramics upon two-step excitation of two-wavelengths. *Sci. Rep.* **7**, 45650; doi: 10.1038/srep45650 (2017).

**Publisher's note:** Springer Nature remains neutral with regard to jurisdictional claims in published maps and institutional affiliations.

## Supplementary Material

Supplementary Information
