# Supplementary material for: Controllable optical modulation of blue/green up-conversion fluorescence from Tm3+ (Er3+) single-doped glass ceramics upon two-step excitation of two-wavelengths
Source: Sci Rep. 2017 Apr 3;7:45650. doi: 10.1038/srep45650 (PMC5377312; doi:10.1038/srep45650)
Supplement: Supplementary Information [file srep45650-s1.pdf]

Supplementary Material for

Controllable optical modulation of blue/green up-conversion fluorescence from Tm^{3+} (Er^{3+}) single-doped glass ceramics upon two-step excitation of two-wavelengths

Zhi Chen,[1†] Shiliang Kang,[1†] Hang Zhang,[2†] Ting Wang,[1] Shichao Lv,[1] Qiuqun Chen,[1] Guoping Dong[1*] and Jianrong Qiu[1,3*]

[1]State Key Laboratory of Luminescent Materials and Devices, and Guangdong Provincial Key Laboratory of Fiber Laser Materials and Applied Techniques, South China University of Technology, Guangzhou 510641, China.

[2]Key Laboratory of Shock Wave and Detonation Physics, Institute of Fluid Physics, CAEP, Mianyang 621900, China.

[3]College of Optical Science and Engineering, State Key Laboratory of Modern Optical Instrumentation, Zhejiang University, Hangzhou 310027, China.

*Correspondence to [dgp@scut.edu.cn (G. Dong), qjr@scut.edu.cn (J. Qiu)]

[†]These authors contributed equally to this work.

1. Theoretical analysis

In this work, we model the dynamics of the fast-slow optical modulation of blue/green fluorescence by means of a master rate equation. This is a reasonable approximation when discussing statistical measurements such as those reported here. We consider three energy levels (1, 2 and 3) as shown in Model 1. The population (N_1, N_2 and N_3) of these levels are assumed to be spin-averaged.[1,2]

1.1 Fast optical modulation

For the fast optical modulation, we built the energy model shown in Model 1a. The dynamics of the system can be ruled by the master rate equation:

$$\frac{dN_1}{dt} = -\omega_{12}N_1 + \omega_{21}N_2 + \omega_{31}N_3$$

(1)

$$\frac{dN_2}{dt} = \omega_{12}N_1 - \omega_{21}N_2 - \omega_{23}N_2$$

(2)

$$\frac{dN_3}{dt} = \omega_{23}N_2 - \omega_{31}N_3$$

(3)

$$N = N_1 + N_2 + N_3$$

(4)

$$\omega_{12} = \alpha I_G \omega_{31}$$

(5)

$$\omega_{23} = \beta I_E \omega_{31}$$

(6)

Where ω_{21} and ω_{31} are intrinsic decay rates, whereas ω_{12} and ω_{23} are excitation rates assumed to be proportional to the GSA wavelength laser (I_G) and ESA wavelength laser (I_E) intensities, respectively; with proportionality constants α and β, expressed in units of the natural decay rate ω_{31}. The steady-state solution of Eq. (1)-(4) is given by

$$N_1 = \omega_{31}(\omega_{21} + \omega_{23})N/[\omega_{31}(\omega_{21} + \omega_{23}) + \omega_{12}(\omega_{31} + \omega_{23})]$$

(7)

$$N_2 = \omega_{12}\omega_{31}N/[\omega_{31}(\omega_{21} + \omega_{23}) + \omega_{12}(\omega_{31} + \omega_{23})]$$

(8)

$$N_3 = \omega_{12}\omega_{23}N/[\omega_{31}(\omega_{21} + \omega_{23}) + \omega_{12}(\omega_{31} + \omega_{23})]$$

(9)

From here, we obtain the fluorescence rate as

$$\mathrm{F} = \eta\omega_{31}N_3 = \frac{\eta\omega_{12}\omega_{23}\omega_{31}N}{[\omega_{31}(\omega_{21}+\omega_{23})+\omega_{12}(\omega_{31}+\omega_{23})]} = \eta\omega_{31}(\frac{\beta I_E N}{1+\beta I_E+\gamma/\alpha I_\mathrm{G}})$$

(10)

Where η is the collection efficiency of the detector, and

$$\gamma = \frac{\omega_{21}}{\omega_{31}} + \beta I_E$$

(11)

1.2 Slow optical modulation

For the slow optical modulation, we built the energy model shown in Model 1b. The dynamics of the system can be ruled by the master equation:

$$\frac{dN_1}{dt} = -\omega_{12}N_1 + \omega_{31}N_3$$

(12)

$$\frac{dN_2}{dt} = \omega_{12}N_1 - \omega_{23}N_2$$

(13)

$$\frac{dN_3}{dt} = \omega_{23}N_2 - \omega_{31}N_3$$

(14)

$$N = N_1 + N_2 + N_3$$

(15)

$$\omega_{12} = \alpha I_G\omega_{31}$$

(16)

$$\omega_{23} = \beta I_E\omega_{31}$$

(17)

Where the corresponding parameters are similar with that shown in the section of fast

optical modulation theoretical analysis. The steady-state solution of Eq. (12)-(15) is given by

$$N_1 = \omega_{31}\omega_{23}N/[\omega_{31}\omega_{23} + \omega_{12}(\omega_{31} + \omega_{23})] \tag{18}$$

$$N_2 = \omega_{12}\omega_{31}N/[\omega_{31}\omega_{23} + \omega_{12}(\omega_{31} + \omega_{23})] \tag{19}$$

$$N_3 = \omega_{12}\omega_{23}N/[\omega_{31}\omega_{23} + \omega_{12}(\omega_{31} + \omega_{23})] \tag{20}$$

From here, we obtain the fluorescence rate as

$$\mathrm{F} = \eta\omega_{31}N_3 = \frac{\eta\omega_{12}\omega_{23}\omega_{31}N}{[\omega_{31}\omega_{23} + \omega_{12}(\omega_{31} + \omega_{23})]} = \eta\omega_{31}(\frac{\beta I_E N}{1 + \beta I_E + \gamma/\alpha I_{\mathrm{G}}}) \tag{21}$$

Where η is the collection efficiency of the detector, and

$$\gamma = \beta I_E \tag{22}$$

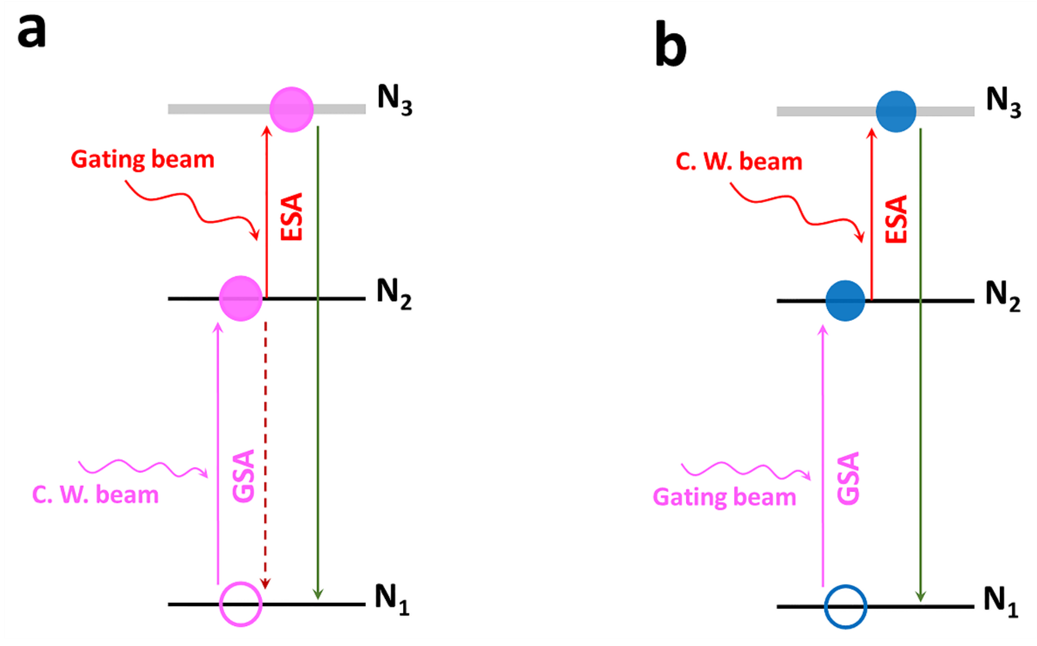

Model 1. Model of energy bands for fast (**a**) and slow (**b**) optical modulation of blue/green UC fluorescence from Tm^{3+} (Er^{3+}) single-doped GCs upon two-step excitation of two-wavelengths, consisting of a ground state 1, an excited state 2 and an excited state 3 for UC fluorescence.

2. Supplementary Data

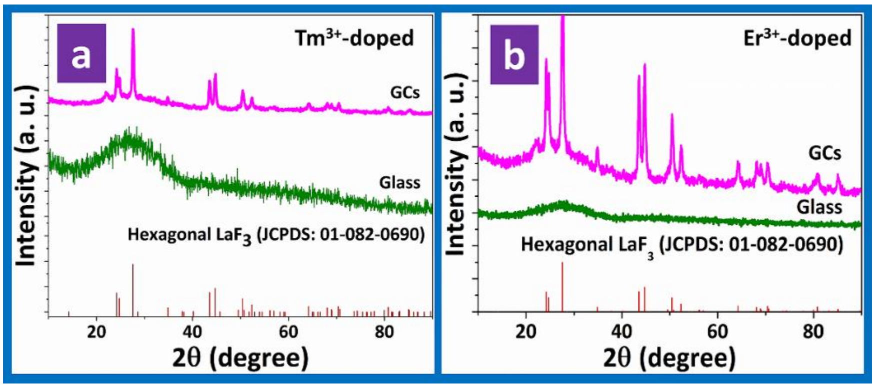

Figure S1: XRD patterns of Tm^{3+} (**a**) and Er^{3+} (**b**) single-doped glass and glass ceramics (GCs) heat-treated at 680 ^{o}C for 4 h. The amorphous solids heat-treated at 680 ^{o}C for 4 h result in the precipitation of LaF_3 nanocrystals (JCPDS [01-082-0690]).

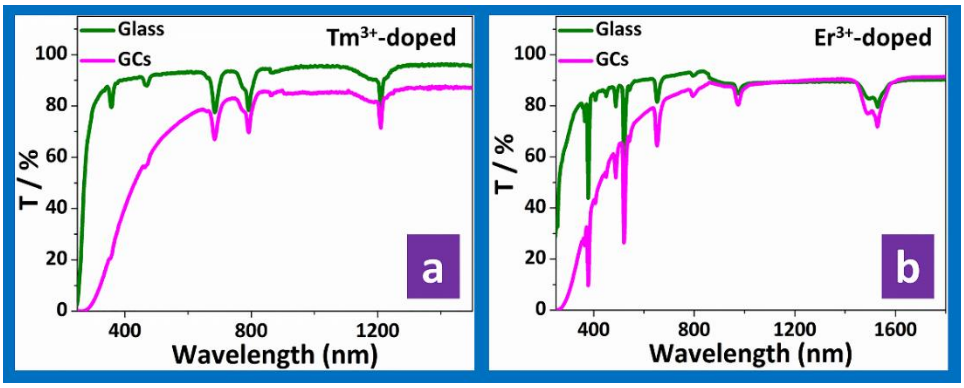

Figure S2: The transmittance spectra of Tm^{3+} (**a**) and Er^{3+} (**b**) single-doped glass and GCs heat-treated at 680 ^{o}C for 4 h. The transmittance spectra show that both of the Tm^{3+} (Er^{3+}) single-doped GCs keep high transparency from visible to near-infrared windows, which guarantee the future photonics application.

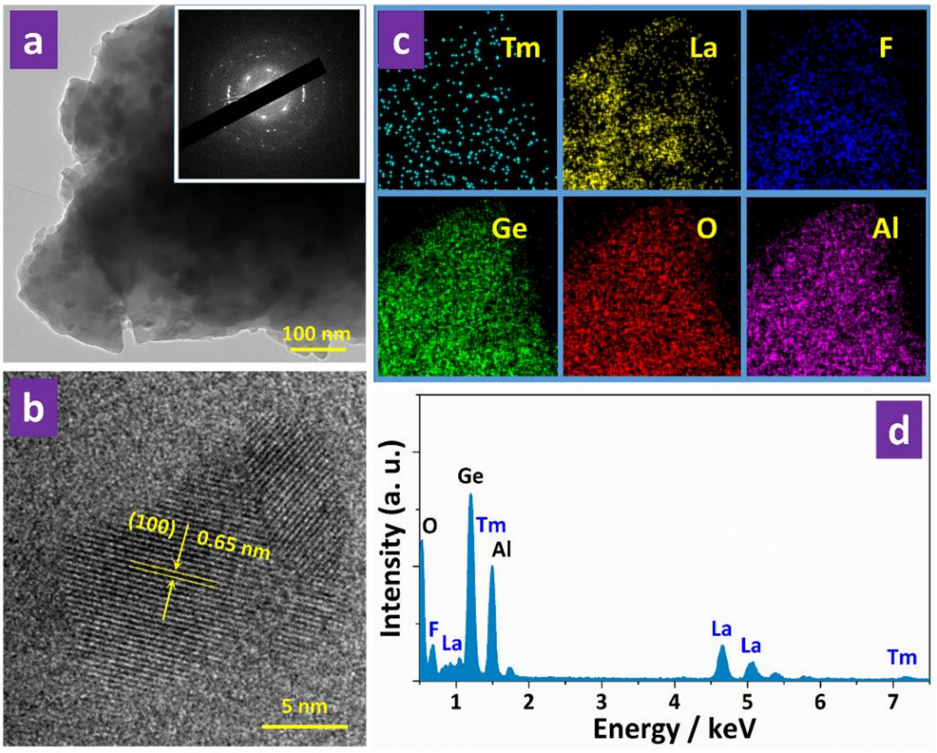

Figure S3: (**a**) TEM images of the Tm^{3+} single-doped GCs heat-treated at 680 °C for 4 h. The inset shows the corresponding selected-area electron diffraction (SAED) pattern. (**b**) high-resolution transmission electron microscope (HRTEM) image of the corresponding nanocrystals in (**a**). (**c**) Two-dimensional mapping distribution images of La, Tm, F, Ge, O, and Al elements of Tm^{3+} single-doped GCs. (**d**) EDX spectrum of the Tm^{3+} single-doped GCs heat-treated at 680 °C for 4 h. The TEM images provide proofs that teat-treatment at 680 ^{o}C for 4 h of the amorphous solids result in the precipitation of nanocrystals with an average size of 16 nm. The SAED rings further validate that the crystalline phase is LaF_3. The HRTEM image clearly display high resolved lattice fringe with constant spacing of 0.65 nm, corresponding to the (100) plane of hexagonal LaF_3 phase. The two-dimensional mapping distribution of elements and EDX results further confirm that Tm^{3+} ions exist as LaF_3 nanocrystals embedded into GCs.

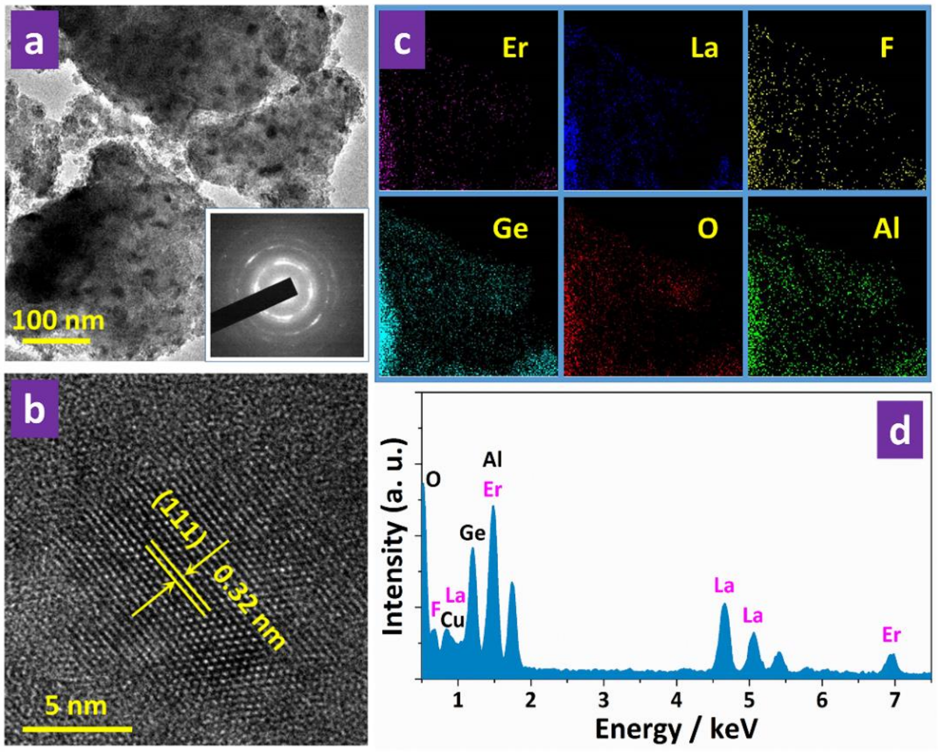

Figure S4: (**a**) TEM images of the Er^{3+} single-doped GCs heat-treated at 680 °C for 4 h. The inset shows the corresponding SAED pattern. (**b**) HRTEM image of the corresponding nanocrystals in (**a**). (**c**) Two-dimensional mapping distribution images of La, Er, F, Ge, O, and Al elements of Er^{3+} single-doped GCs. (**d**) EDX spectrum of the Er^{3+} single-doped GCs heat-treated at 680 °C for 4 h. The TEM images provide proofs that teat-treatment at 680 ºC for 4 h of the amorphous solids result in the precipitation of nanocrystals with an average size of 16 nm. The SAED rings further validate that the crystalline phase is LaF_3. The HRTEM image clearly display high resolved lattice fringe with constant spacing of 0.32 nm, corresponding to the (111) plane of hexagonal LaF_3 phase. The two-dimensional mapping distribution of elements and EDX results further confirm that Er^{3+} ions exist as LaF_3 nanocrystals embedded into GCs.

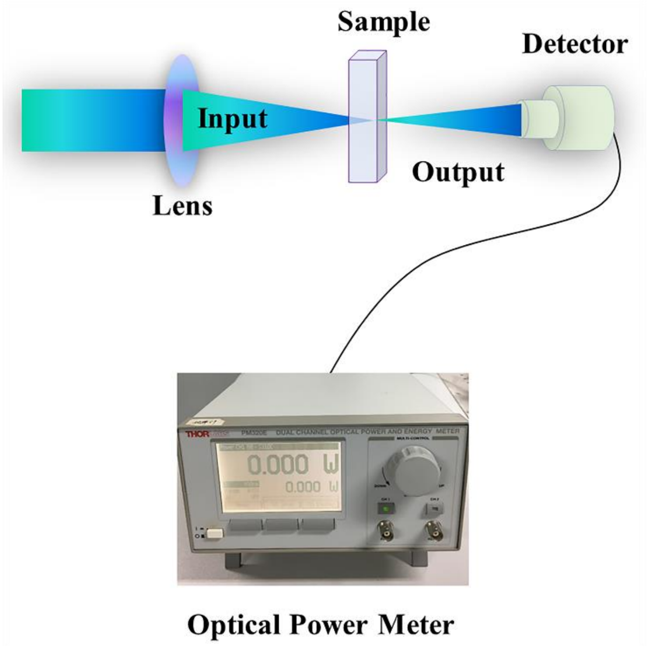

Figure S5: Home-built optical setup for the measurement of optical loss of the well-engineered GCs single-doped with Tm^{3+} or Er^{3+}. The optical loss (α) of the GCs can be defined as following:

$$\alpha = -\frac{10 \log \frac{B}{A}}{l} \quad (1)$$

Where A denotes the laser input power, B presents the laser output power passing through the GCs sample, l is the GCs sample thickness. As two wavelengths lasers are involved in our pumping strategy for fast-slow optical modulation, we measured the optical losses at 800 and 1064 nm for Tm^{3+} single-doped GCs, and that at 850 and 1530 for Er^{3+} single-doped GCs, which are summarized in **Table S1**.

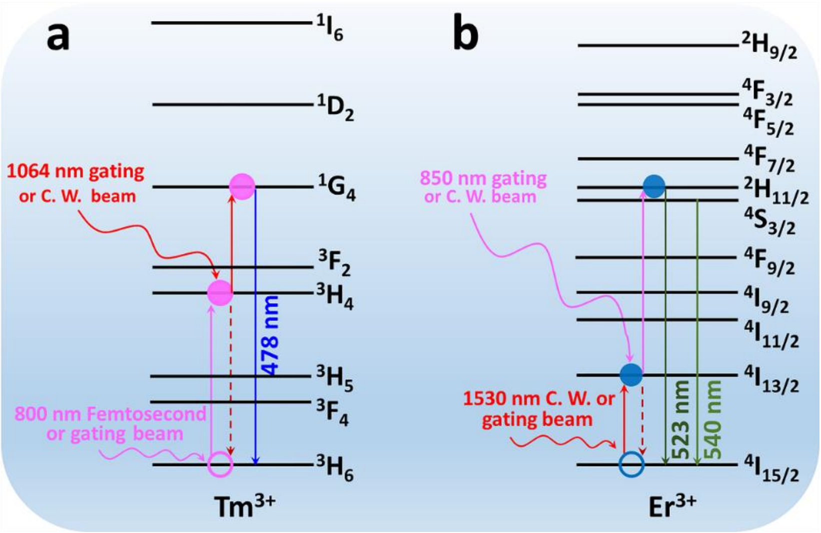

Figure S6: Energy levels schematic of Tm^{3+} (a) and Er^{3+} (b) single-doped GCs upon two-step excitation of two-wavelengths. Under this pumping tactic, no other ESA processes are involved in the fast-slow optical modulation of blue/green UC fluorescence.

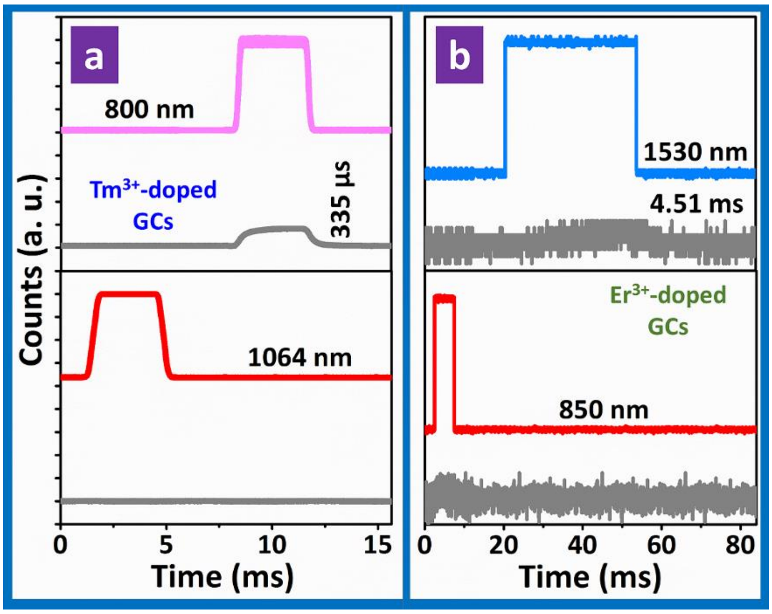

Figure S7: Time-dependent fluorescence of Tm^{3+} (**a**) and Er^{3+} (**b**) single-doped GCs following repeated pulse sequence of only single-wavelength laser light pulse. No or negligible fluorescence signal is detected with only single-wavelength irradiation. For Tm^{3+} single-doped GCs, no fluorescence signal is detected with only 1064 nm excitation, and only 800 nm illumination leads to negligible fluorescence signal with response time of 335 μs. For Er^{3+} single-doped GCs, only 1530 nm illumination can lead to negligible fluorescence signal with response time of 4.51 ms, and no fluorescence signal is detected with only 850 nm excitation.

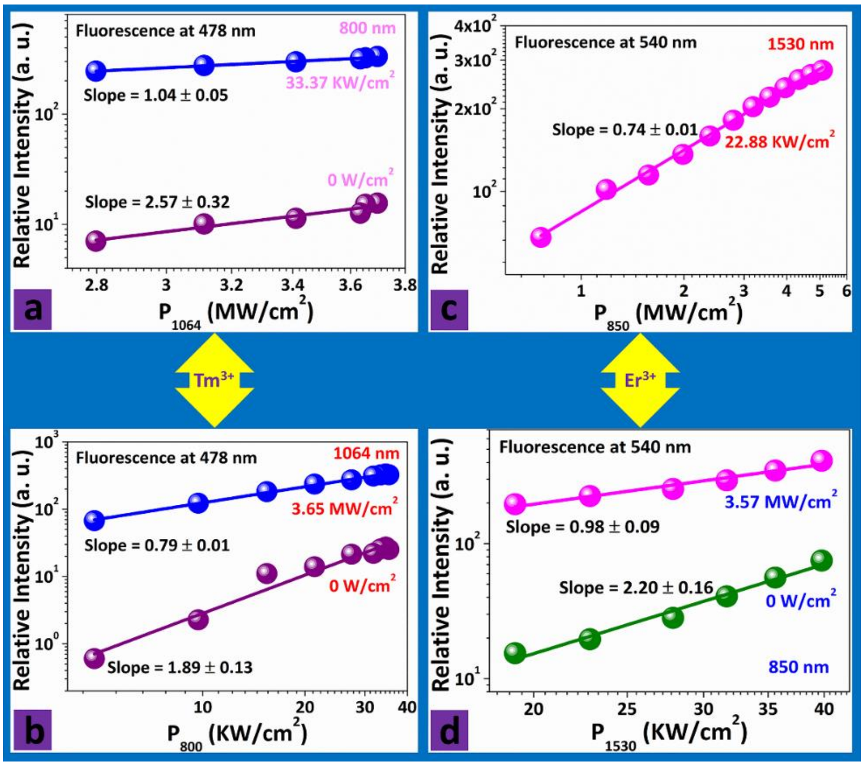

Figure S8. Double logarithmic dependence of the blue/green UC fluorescence from Tm^{3+} (**a, b**) and Er^{3+} (**c, d**) single-doped GCs on the laser powers of the single-wavelength excitation or two-step excitation of two-wavelengths with one laser power fixed. The number of photons required for the UC fluorescence yield can be obtained from the formula: $I_{em} \propto P^n$, where I_{em} is the emission intensity, P is the pump laser power, and n is the number of laser photons.[3-5] The NIR laser power dependence of the fluorescence counts shows an apparently linearity relationship with increasing NIR laser power before reaching saturation. Under two-step excitation of two-wavelengths, only one photon is required for the blue/green UC fluorescence, which indicates the existing of an effective ESA process. However, two or three photons are needed for producing the blue/green UC fluorescence only upon single-wavelength excitation.[6,7] The results hint that the speed of electrons populated fully in the excited state can be tuned by various pumping methods, namely C. W.

laser of GSA wavelength coupled with gating laser of ESA wavelength for the electrons fast populating in the excited state fully, or C. W. laser of ESA wavelength coupled with gating laser of GSA wavelength for the electrons slowly populating in the excited state fully.

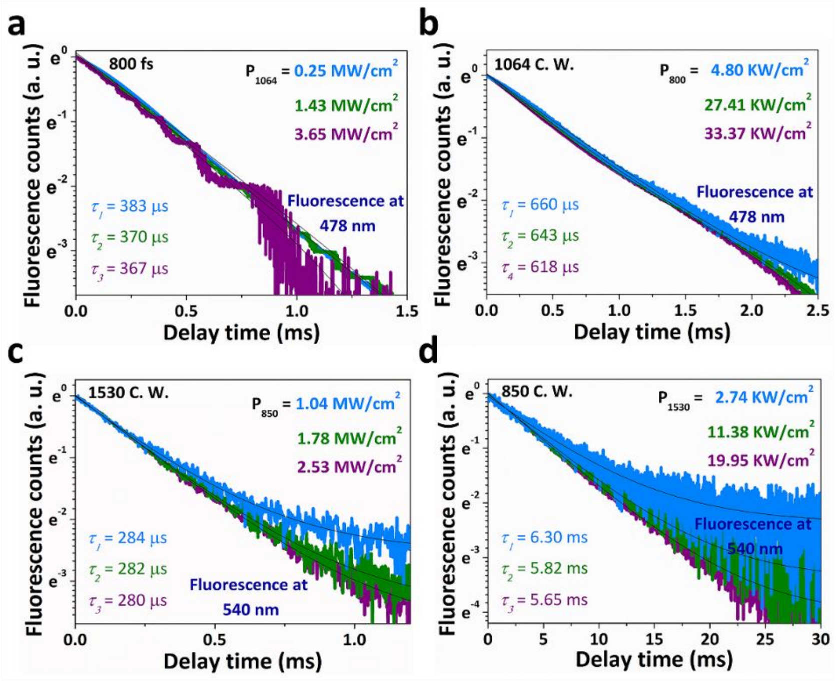

Figure S9: The measured power dependence of the blue/green UC fluorescence lifetime performed on Tm^{3+} (**a, b**) and Er^{3+} (**c, d**) single-doped GCs after excitation of one gating NIR laser combined with another C. W. laser illumination is extracted from the fits. The fixed C. W. laser power of 800 nm (**a**), 1064 nm (**b**), 1530 nm (**c**), and 850 nm (**d**) is 33.37 KW/cm^2, 3.65 MW/cm^2, 11.38 KW/cm^2, 2.53 MW/cm^2, respectively. The effect of the NIR gating laser is best observed through the reduction with increasing its power that it produces in the lifetime of the excited state for blue/green UC fluorescence generation whether in Tm^{3+} (Er^{3+}) single-doped GCs.

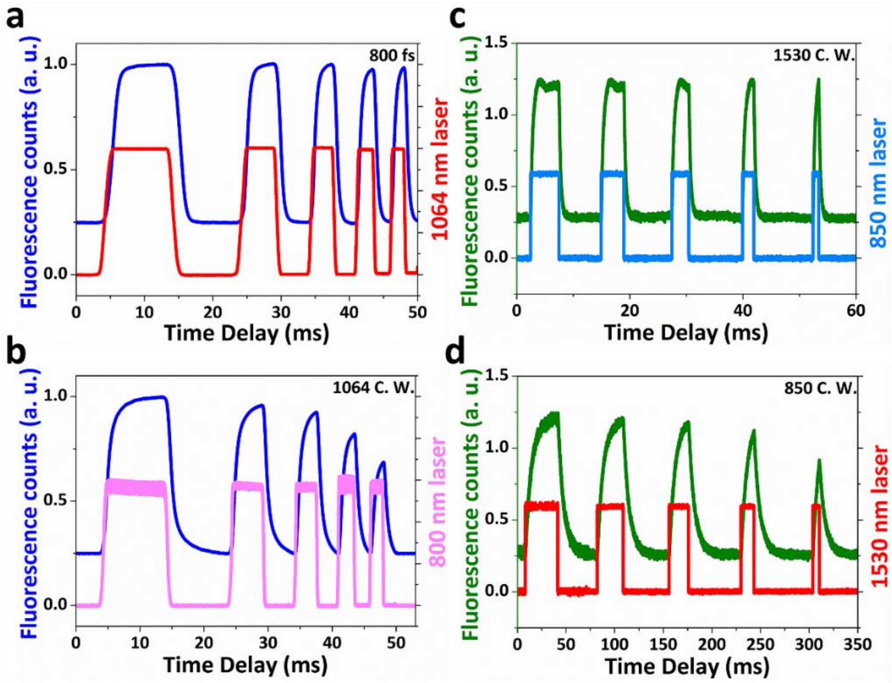

Figure S10. The measured blue/green fluorescence from Tm^{3+} (**a, b**) and Er^{3+} (**c, d**) single-doped GCs for different gating laser pulse width of one NIR laser beam coupled with another C. W. laser beam illustrating the time response of the system. When the GSA wavelength laser is tuned as C. W. signal, the ESA wavelength laser can effortlessly and quickly pump the electrons populated in the first excited state to the UC fluorescence level for getting a steady-state despite we use the shortest pulse width, resulting in an unlimited UC fluorescence decay from steady-state to ground state through radiation transition. Instead, the electrons populated in the first excited state cannot be rapidly pumped by the ESA wavelength laser to the UC fluorescence level for getting a steady-state with the pulse width diminished when the GSA wavelength laser is tuned as gating signal, leading to the UC fluorescence decay from non-steady-state to ground state through radiation transition.

Table S1. Summary of the optical loss of Tm^{3+} single-doped GCs measured at continuous-wave laser at 800 and 1064 nm, and that of Er^{3+} single-doped GCs measured at continuous-wave laser at 850 and 1530 nm, respectively.

Samples	Wavelengths (nm)	Thickness (mm)	Input power (mw)	Output power (mw)	Optical loss (dB/cm)
Tm^{3+} doped	800	1.16	83.53	50.32	12.87
GCs	1064	1.16	85.53	74.11	3.64
Er^{3+} doped	850	1.71	80.46	67.01	4.65
GCs	1530	1.71	79.11	57.23	8.22

From the results of the measured optical losses we can clearly observed that, the GCs at the laser used for GSA has higher optical loss in comparison with that for ESA.

References

1 Deng, R. *et al.* Temporal full-colour tuning through non-steady-state upconversion. *Nat. Nanotechnol.* **10**, 237-242 (2015).

2 Rodríguez-Rodríguez, H., Imanieh, M., Lahoz, F. & Martín, I. Analysis of the upconversion process in Tm^{3+} doped glasses for enhancement of the photocurrent in silicon solar cells. *Sol. Energy Mater. Sol. Cells* **144**, 29-32 (2016).

3 Dong, H. *et al.* Photon upconversion in Yb^{3+}–Tb^{3+} and Yb^{3+}–Eu^{3+} activated core/shell nanoparticles with dual-band excitation. *J. Mater. Chem. C* **4**, 4186-4192 (2016).

4 Wu, M. *et al.* Single-band red upconversion luminescence of perovskite $KMgF_3$: Yb/Er nanocrystals and its enhancement via Mn^{2+} doping. *J. Mater. Chem. C* **4**, 1675-1684 (2016).

5 Yang, Y. *et al.* $NaYF_4$: Yb^{3+}, Tm^{3+} inverse opal photonic crystals and $NaYF_4$: Yb^{3+}, Tm^{3+}/TiO_2 composites: synthesis, highly improved upconversion properties and NIR photoelectric response. *J. Mater. Chem. C* **4**, 659-662 (2016).

6 Chen, X. *et al.* Large Upconversion Enhancement in the "Islands" Au–Ag Alloy/$NaYF_4$: Yb^{3+}, Tm^{3+}/Er^{3+} Composite Films, and Fingerprint Identification. *Adv. Funct. Mater.* **25**, 5462-5471 (2015).

7 Guo, H. *et al.* Visible upconversion in rare earth ion-doped Gd_2O_3 nanocrystals. *J. Phys. Chem. B* **108**, 19205-19209 (2004).
